# Weed biodiversity and herbicide intensity as linked via a decision support system

**DOI:** 10.1002/ps.70019

**Published:** 2025-07-29

**Authors:** Friederike de Mol, Robert Fritzsche, Bärbel Gerowitt

**Affiliations:** ^1^ Faculty of Agricultural and Environmental Sciences, Crop Health University of Rostock Rostock Germany

**Keywords:** herbicide decision, path model, species richness, Shannon index, weed density, winter wheat

## Abstract

**BACKGROUND:**

Extensive herbicide use is one reason for the declining biodiversity of arable weeds. This study aimed to investigate (i) whether herbicide decisions recommended by a decision support system increase the weed species diversity compared to standard recommendations, and (ii) whether high weed species diversity reduces herbicide intensity, which in turn contributes to higher diversity. Data on weeds and herbicide applications in winter wheat fields in north‐eastern Germany were collected in 15 field trials over 2 years. Five treatments differed in the way of decision‐making for herbicide application, including two treatments according to recommendations of decision support systems.

**RESULTS:**

Along the Hill's series biodiversity metrics, the untreated control had the highest species richness (13.5 m^−2^) per field but showed increasingly stronger dominance structures than the treated plots (equivalent species richness: 1.7–2.0 m^−2^). The treatment frequency index as a metric for herbicide intensity was significantly lowest in the decision support system with low reliability (1.07). Path models, including weed diversity and density in autumn, weed diversity in summer, and herbicide intensity as a mediating variable showed a significant decreasing effect of Shannon diversity on herbicide intensity in all treatments. Only the decision support systems reacted to low weed densities with a significant reduction of the herbicide intensity.

**CONCLUSION:**

Higher weed species diversity contributes to lower herbicide intensity, which is ecologically and economically valuable. Decision support systems for herbicide application should have other target functions than cost reduction for contributing to biodiversity. © 2025 The Author(s). *Pest Management Science* published by John Wiley & Sons Ltd on behalf of Society of Chemical Industry.

## Graphical abstract

A high Shannon diversity of weed species leads to low herbicide intensity. The decision support system reduces herbicide intensity at low weed densities and thus supports biodiversity.

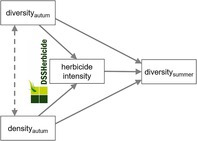

## 1 INTRODUCTION

The biodiversity of weeds on conventionally cultivated fields has decreased over the last decades.^1^, ^2^, ^3^, ^4^ Preserving and promoting weed diversity is desirable because weeds provide regulating ecosystem services in the agro‐ecosystem.^5^ In conventional farming, herbicides play a key role in controlling weeds. While the control effects are intended, herbicide use is a major driver of biodiversity loss in arable weeds.^6^ However, Storkey and Westbury proposed the use of selective herbicides to promote biodiversity.^7^ In particular, highly competitive monocotyledonous species should be suppressed to allow diversity in the dicotyledonous species. Species management through selective herbicides was possible for some regionally endangered species, such as the corn poppy *Papaver rhoeas* L. and cornflower *Centaurea nigra* L.^8^ Nevertheless, Storkey and Westbury and Ulber *et al*. judged that, generally, herbicides did not act specifically enough to target weed species and their species composition in management.^7^, ^8^

However, it may be possible to promote biodiversity without specifying target species. This requires an excellent knowledge of the dose–response relationships of individual herbicides and herbicide mixtures. The decision support system (DSS) Crop Protection Online from Denmark (CPO) for optimizing herbicide use provides this knowledge.^9^, ^10^ An optimization algorithm should assist in selecting the herbicides and their dosages, as the multiple‐species–multiple‐herbicide optimization problem can probably be better solved by computers rather than by humans. The algorithm of CPO aims to minimize costs while maintaining a constant yield level. Dose–response relationships for each herbicide and weed species combination, the minimum control success (‘target efficacy’) to be achieved for each weed species in terms of its density, and stage of development are included in CPO as a data basis. Prototypes of CPO have been successfully tested in several European countries, including Germany, Norway, Poland, the Baltic states, and Spain.^11^, ^12^, ^13^ Although the aim of the CPO is to optimize herbicide use economically, reducing herbicide intensity is also often achieved.^13^ However, reduced herbicide intensity can, in turn, promote greater weed diversity by lowering the selection pressure on susceptible species.^14^, ^15^ CPO particularly targets dominant species due to their species characteristics and their densities. By controlling these dominant species, a changed composition of weed species and a more even distribution of species is expected, resulting in more biodiversity, as determined by, for example, the Shannon index.

Generally, the negative effect of herbicide intensity on weed diversity is not questioned today.^16^, ^17^, ^18^ However, the reverse effect can also be stated: can higher weed diversity reduce herbicide intensity without sacrificing yield? This refers to the question posed by Gerowitt *et al*. with the ‘A for B’ and ‘B for A’ approach in the context of functional biodiversity: How does agriculture (‘A’) support biodiversity (‘B’) and how does biodiversity (‘B’) support agriculture (‘A’)?^19^ Studies by Storkey and Neve and Adeux *et al*. revealed that the yield loss caused by weeds decreased with more diverse weed communities, thus supporting the ‘biodiversity supports agriculture’ hypothesis for weeds.^20^, ^21^

In a review article, Blaix *et al*. write that high weed diversity improves several regulating ecosystem services weeds provide.^5^ However, the authors do not mention the need for weed control as a regulating service, so it can be assumed that this topic has not been intensively researched. To the best of our knowledge, the influence of weed species diversity on herbicide intensity has not yet been conclusively clarified in field trials or in weed studies.

We conducted 15 field trials in 2 years in winter wheat fields in north‐eastern Germany to test whether recommendations for a herbicide DSS were suitable for promoting weed biodiversity without the loss of yield and profit. We followed the suggestions of a German prototype of the Danish CPO. Dose–response relations were adjusted to German conditions, and the target efficacies were defined in two DSS versions: a standard version, similar to the Danish template, and a riskier version with reduced target efficacies. An untreated control and herbicide treatments applied as decided by weed and herbicide experts served as references in the field experiments. Testing the effect of weed species diversity on herbicide intensity in field trials requires a herbicide choice based on objective criteria. DSSs for optimizing herbicide use offer this objectivity. Therefore, the winter wheat trials with recommendations following a DSS also allowed us to explore the mutual relationship between weed biodiversity and herbicide intensity. We developed path models to identify and quantify the agricultural and ecological interrelationships, using weed density as an additional influencing variable.

Weed diversity, weed density and herbicide use form a system where the individual components influence each other. The objective of this study was to describe the relation between weed infestation and herbicide intensity and how different decision‐making processes influence them. We hypothesize that.

- using a DSS can contribute to conserving biodiversity and reducing pesticide intensity through the targeted choice of herbicides,
- increasing biodiversity reduces weed herbicide intensity, and in turn, reduced herbicide intensity sequentially contributes to higher weed biodiversity.

## 2 MATERIAL AND METHODS

### 2.1 Field trials

**Figure 1:**
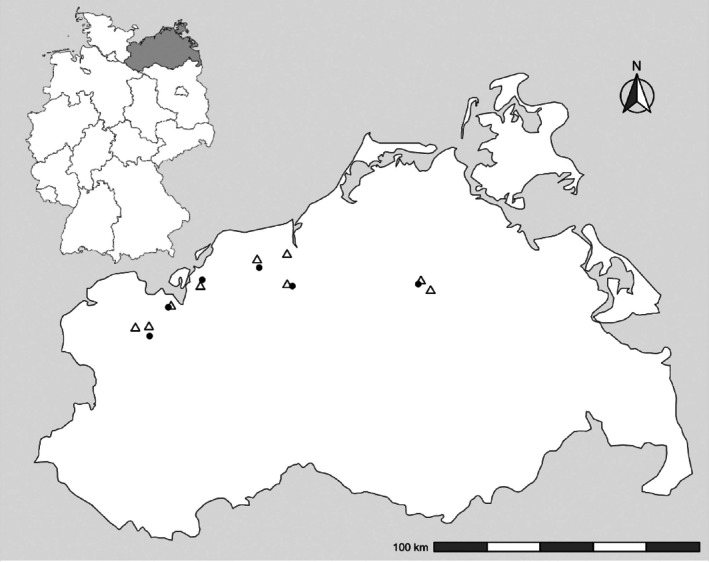
Field trial locations. top left: Germany with the north‐eastern region (grey); main map: north‐eastern region with trial locations in the years 2011–2012 (●) and 2012–2013 (Δ).

In 2011–2012 and 2012–2013, 15 herbicide field trials identically designed were carried out in north‐eastern Germany, six in the first and nine in the second experimental year (Fig. 1). The sites were on conventional winter wheat fields with low‐loamy to high‐loamy, sandy soils. Before sowing, all fields were ploughed with a chisel or a moldboard plough, in accordance with the usual soil cultivation. The average long‐term precipitation in the region is 615 mm, and the average annual temperature is 9.1 °C.^22^

The field trials were carried out according to the European and Mediterranean Plant Protection Organization (EPPO) standards as a randomized block design with four replicates.^23^, ^24^ Trial plots were 2.5 m wide in all trials; plots lengths depended on the farmer's equipment, resulting in plot areas of 18.75, 25.0, and 26.25 m^2^. The decision‐making process for herbicide use was the only experimental factor, which had five levels:

1. DSSstd: Decisions according to a regional prototype of the CPO system.^13^ The prototype assessed data of 21 herbicides and 21 weed species.
2. DSSred: Equal to DSSstd, but reduced target efficacies by 3% to 15%, depending on the weed species.
3. AdvLoc: Recommendation of a local agricultural advisor; the field was inspected before being recommended. The advisors were bound to the DSS herbicide portfolio.
4. AdvSer: Standard recommendations of the official advisory service. Fields were not inspected. All approved herbicides were allowed.
5. Untreated: Untreated control without herbicide spraying.

The herbicides were applied by a professional field trial company, while the research team assessed the weeds. Approximately 4 weeks after the application, efficacies were assessed; a follow‐up treatment was applied if the effect had been insufficient. Herbicides were used strictly according to their authorization (quantities, season and stage of development). Within a field, the treatments were carried out on the same day. At the first treatment in autumn, the winter wheat had 1–3 leaves. If further herbicide applications were required, they were carried out in the autumn at the start of tillering of the wheat and/or at the start of stem elongation in the spring. All other crop management practices were carried out by the farmer in accordance with good agricultural practices, consistent with the surrounding fields.

### 2.2 Weed surveys

Weed surveys analysed in this article took place in autumn and close to harvest.

In autumn, prior to any control, species‐specific weed densities were counted per block in ten irregularly distributed 0.1 m^2^ squares. After the weed assessment, the treatment plots were randomized within the blocks to avoid bias due to inhomogeneous weed occurrence. Together with the development stage of the weeds, this survey was used as the basis for the control decision in autumn. Treated plots were checked again in spring (5 × 0.1 m^2^ per plot) to decide about an additional spring weed control.

In early summer, all herbicide treatments were completed. At the winter wheat milk ripeness stage, weed dry biomass was assessed by cutting all weeds in 1 m^2^ per plot at ground level, separating the fresh mass by species, drying it at 60 °C to a constant weight, and then weighing it.

Weed data was aggregated separately by species but pooled per treatment and location across the blocks for the herbicide decisions and for further data processing.

### 2.3 Data processing and statistical analysis

For the description of weed diversity, the Rényi diversities were calculated along the Hill series with Hill's power *a* equal to 0, 0.5, 1, 2, 4 and infinity.^25^, ^26^

$${\text{diversity}}_{a}={\left(\sum_{i=1}^{S}\left(p_{i}^{a}\right)\right)}^{1/\left(1-a\right)}$$

with *p*_*i*_ being the proportion of individuals belonging to species *i* and *S* the total number of species.

The calculation with Hill's power *a* = 0 corresponds to the richness (= species number), with *a* = 1 to the equivalent species richness (= true diversity, = exponent of the Shannon index), with *a* = 2 to the inverse Simpson index and with *a* = infinity to the reciprocal of the Berger–Parker index.^25^ For the richness and the equivalent species richness, the *γ*‐diversity for the total of the experimental fields and the *α*‐diversity for the mean of the individual fields were calculated according to Whittaker.^27^ The other diversity metrics were calculated at the experimental field level. Diversity metrics for the autumn weed survey are based on plant counts, while for the early summer survey, they are based on the dry biomass of the weeds.

Herbicide intensity was calculated as treatment frequency index (TFI).^28^ TFI adds up the applied dosage in relation to the registered dosage for all herbicides within the cropping year.

All statistical analyses were performed in the software R.^29^

The treatment effects on TFIs, weed biomass, and weed diversity metrics were analysed using exact all‐pairs comparison tests of Friedman‐type ranked data. For this, experimental fields were regarded as replications. The tests were performed according to Eisinga *et al*. without *P*‐adjustment as they are implemented in the R‐package ‘PMCMRplus’.^30^, ^31^

**Figure 2:**
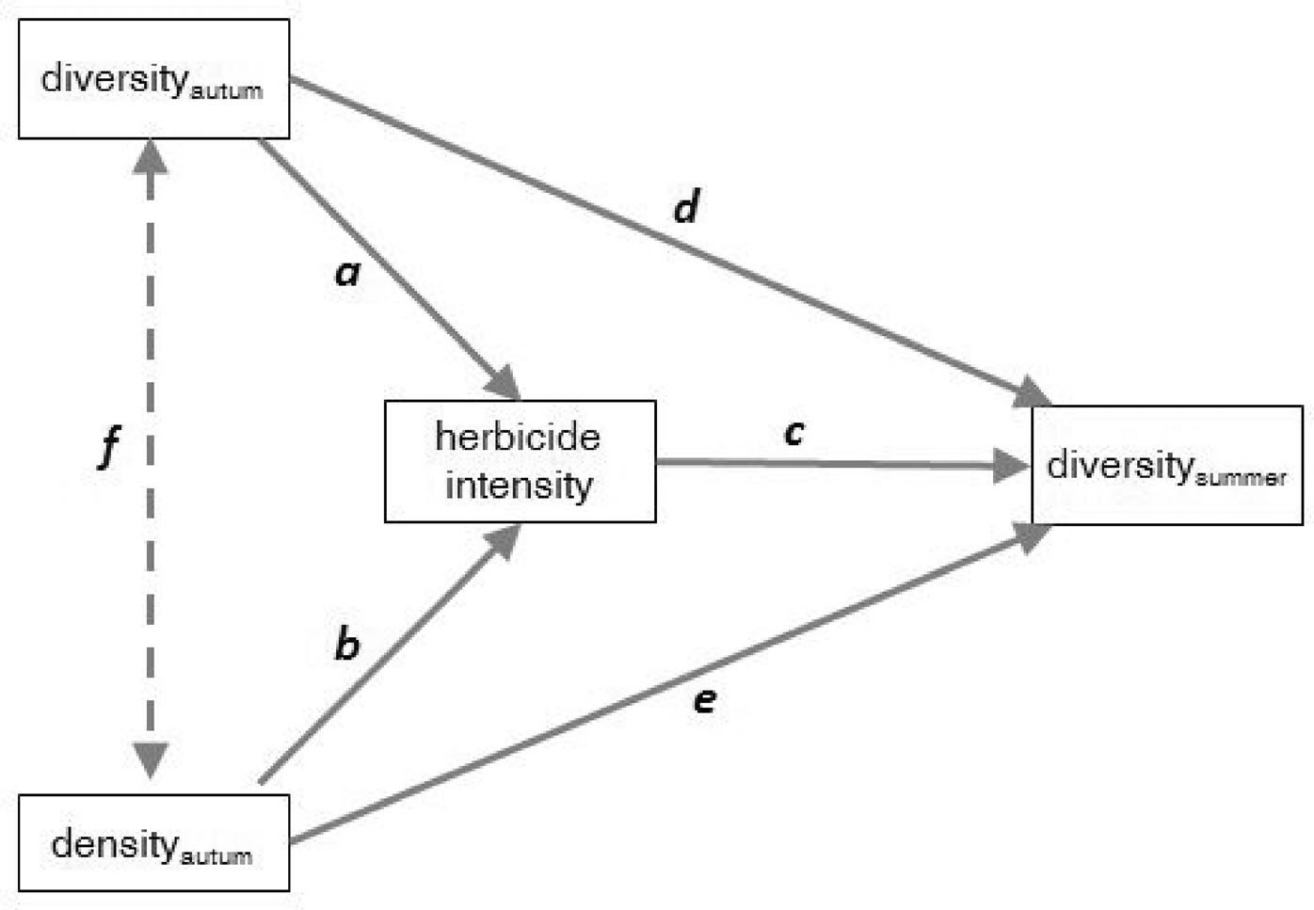
Graphical representation of a path model on the mutual influences of weed diversity and herbicide intensity, including weed density. Solid arrows a–e: regressions (explanatory variable at the start point explains response variable at the end point of the arrow); dashed arrow f: correlation (end point variables are related).

Two path models with the same structure were set up, one with species richness and one with the Shannon index (= logarithm of the equivalent species richness) as a diversity metric (see Fig. 2 for a graphical representation of the models). By means of the path models, the hypotheses were tested that biodiversity in autumn influenced TFI (path a), which in turn influenced diversity later in the year (path c). The algorithm of the DSS led to higher targeted effects for higher species densities, which subsequently required higher dosages. Therefore, weed density in autumn was included as an additional independent variable (path b). Two regressions were introduced to model how density and diversity in autumn directly affected diversity in early summer (paths d and e). Correlation f assumed that weed density and diversity in autumn correlated (Fig. 2). Only data from treated plots were considered in the path model. Weed densities in autumn were log‐transformed to reduce the strong leverage of high densities. The regression parameters were initially estimated as a multi‐group model, that is separate parameters were adjusted for each treatment. Stepwise, single parameters were constrained to be equal across groups. After each step a likelihood‐ratio test for comparing the nested models was performed. The model selection (i.e., comparisons between nested models) results in the unstandardized coefficients in natural units. Additionally, we present the standardized coefficients, which are appropriate for a comparison within models. The R package ‘lavaan’ was used as software for the path modelling and the likelihood‐ratio tests.^32^

The impact of the herbicide treatments on the species composition was studied using PERMANOVA (permutational multivariate analysis of variance). The experimental fields were taken as constraining strata. Two PERMANOVAs were run with 500 permutations each. They differed in including/excluding the untreated control. An RDA (redundancy analysis) ordination on Hellinger‐transformed species biomass data with the treatment as explaining variable and the field as conditioning variable graphically represented the weed composition. Multivariate analyses were done with the R‐package ‘vegan’.^33^

## 3 RESULTS

### 3.1 Weed species diversity metrics and herbicide intensity

**Figure 3:**
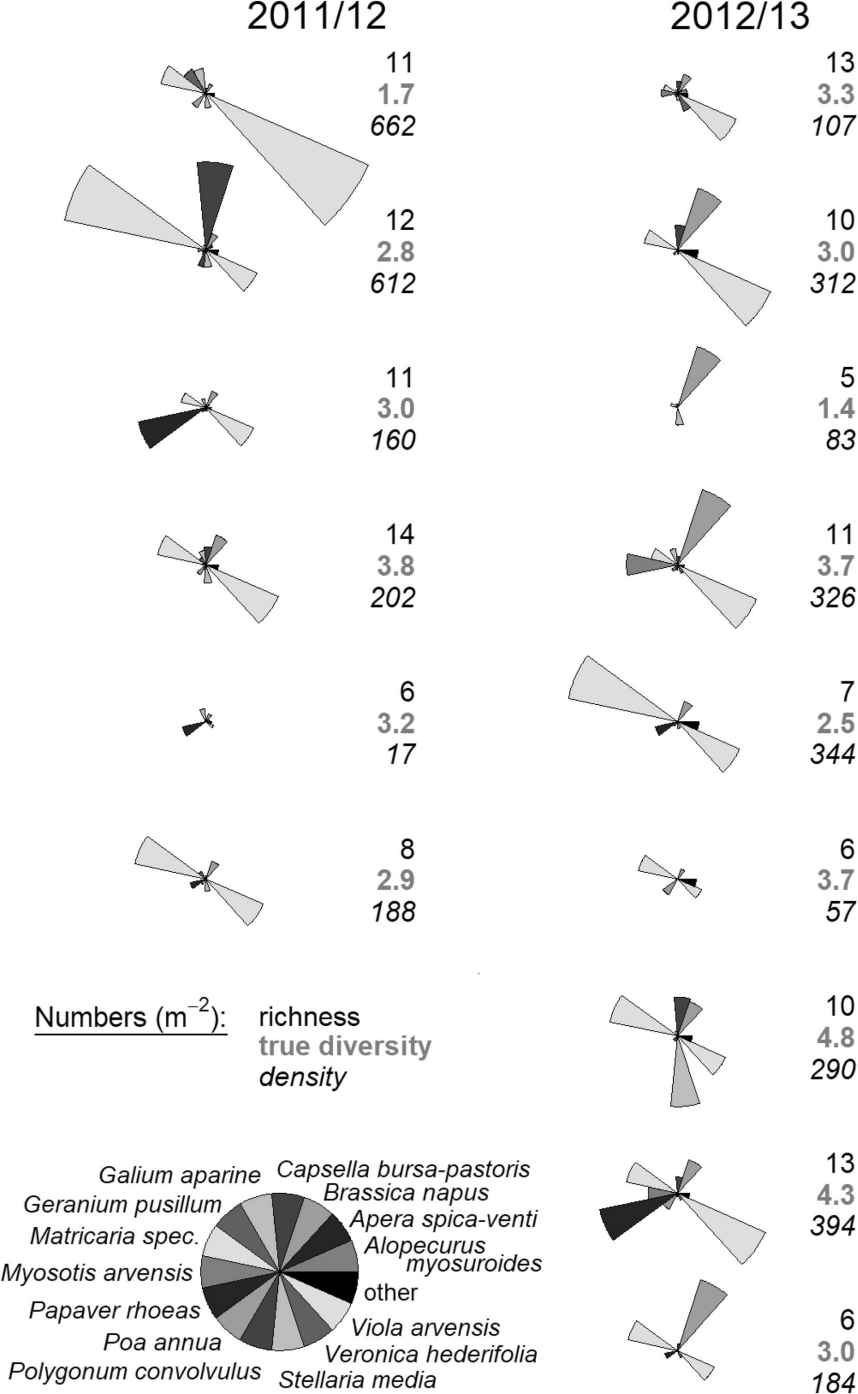
Weed species composition, richness, true diversity (= exp(Shannon index)) and density (m^−2^; means per field) on 15 winter wheat experimental fields in the cropping years 2011–2012 and 2012–2013. Weeds were counted in autumn before weed management at a wheat development stage of one to three leaves. Weed counting was block wise (four blocks) before the set‐up of treatment plots. Species densities are proportional to the segment areas in the star plots.

Weed densities in autumn ranged from 17 to 662 plants m^−2^ (Fig. 3). Species richness varied from 6 to 14, and the true diversity (exponent of the Shannon index) from 1.4 to 4.8 (Fig. 3). Species were predominantly dicotyledonous with high amounts of *Viola arvensis* Murr., *Matricaria* spec. and *Papaver rhoeas* L. (Fig. 3). In some sites, volunteer oilseed rape was an important part of the weed vegetation.

The different ways of decision‐making resulted in graduated herbicide intensity (Table 1, TFI); the treatment according to the AdvSer had the highest TFI at 1.53. The DSSred treatment, with a TFI reduction of one‐third, had the lowest intensity (1.07) and was the only one that differed significantly from the others. DSSstd and AdvLoc treatments had similar total herbicide intensities. All control options significantly reduced weed biomass by 88% to 94% compared to the untreated control (Table 1, Weed biomass). Weed biomass differed not significantly between the two DSS treatments and that of the AdvLoc but was significantly higher than the AdvSer treatment.

**Table 1:** TFI (treatment frequency index) for herbicides in one cropping year, and weed biomass (dry mass) at wheat milk ripeness.

Treatment	Abbreviation	TFI		Weed biomass		
		(%)	Rank	(g m^−2^)	(%)	Rank
Untreated				159.1	100	5.0a
DSS standard	DSSstd	1.37	2.7a	16.3	10	3.0b
DSS with reduced target efficacy	DSSred	1.07	1.4b	14.1	9	2.8b
Local advisor	AdvLoc	1.40	2.9a	19.1	12	2.8b
Advisory service	AdvSer	1.53	3.0a	9.3	6	1.5c

Mean values and mean ranks of 15 winter wheat field trials after herbicide treatment due to different herbicide decision making. Letters indicate homogeneous subgroups (*α* = 5%) according to exact all‐pairs comparisons tests of Friedman‐type ranked data.

DSS, decision support system.

In early summer, a total of 42 species (*γ*‐diversity) and a mean of 13.5 species per field experiment (*α*‐diversity) were identified in the untreated plots (Table 2). Weed control based on DSS decisions and AdvLoc recommendations reduced the number of species by approximately a quarter, both overall and on average across individual fields. Treatments according to the AdvSer approximately halved the species number of the untreated plots. The untreated plots were markedly more diverse than all treated plots in terms of equivalent species richness, followed by the DSSred treatment (Table 2). The equivalent species richness of all treatments was four to five times smaller than the species richness, indicating a strong unequal species distribution.

**Table 2:** Weed *γ*‐ and *α*‐diversity in 15 winter wheat field trials after herbicide treatment due to different herbicide decision making.

Treatment	Abbreviation	Richness		Equivalent richness	
		*γ*	*α*	*γ*	*α*
Untreated		42	13.5	9.9	3.2
DSS standard	DSSstd	28	9.0	4.8	1.8
DSS with reduced target efficacy	DSSred	30	9.2	6.4	2.0
Local advisor	AdvLoc	29	9.5	5.7	1.8
Advisory service	AdvSer	24	5.9	3.9	1.7

Diversity metrics are based on aboveground weed dry mass. Diversity is expressed as species richness and equivalent species richness (= true diversity = exponent of the Shannon index).

DSS, decision support system.

**Figure 4:**
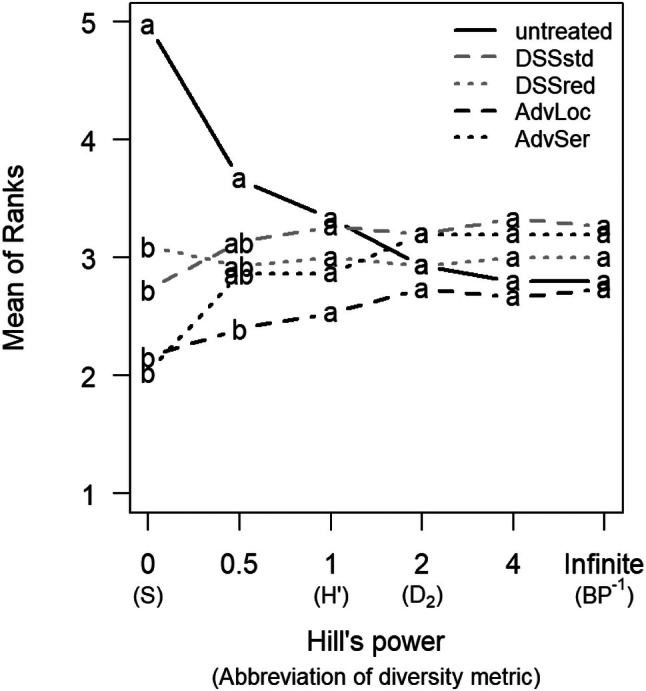
Diversity indices of weed communities along the Hill series. Indices are based on weed biomass (dry mass) of 15 winter wheat field trials, herbicides treated due to different decision making. DSSstd, decision support system standard; DSSred, decision support system with reduced target efficacy; AdvLoc, local advisor; AdvSer, advisory service; *S*, species richness; *H*′, Shannon entropy; *D*_2_, inverse Simpson index; BP^−1^, inverse Berger–Parker index.

Of all the diversity metrics examined in the Hill series, solely species richness differed significantly between the experimental treatments (Fig. 4). The untreated plots had the highest species richness, followed by the plots treated according to the two DSSs. Along the Hill series the mean ranks of the untreated control decreased strongly, while they increased in the treatments DSSstd, AdvLoc and in AdvSer.

### 3.2 Path model results

#### 3.2.1 Different ways of decision making (unstandardized coefficients)

**Figure 5:**
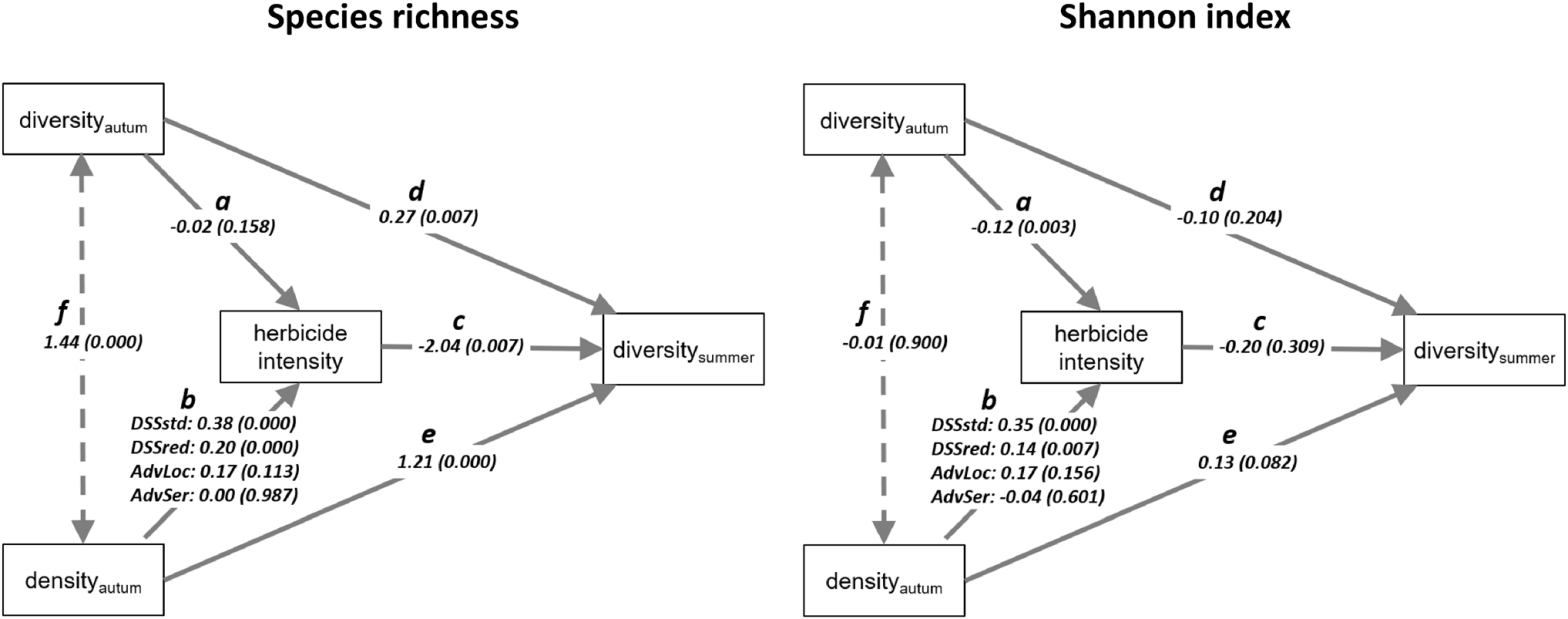
Weed diversity–herbicide intensity path model: direct effects are presented by path model coefficients (*P*(*z*)‐values). Data are based on 15 winter wheat field trials with different herbicide decision making. DSSstd, decision support system standard; DSSred, decision support system with reduced target efficacy; AdvLoc, local advisor; AdvSer, advisory service. Diversity in autumn is based on counts, diversity in summer after herbicide treatments is based on biomass. Herbicide intensity was determined as treatment frequency index (TFI). Paths a–f show direct effects from the start point to the end point of the arrows. Paths a–f refer to Table 3 and to the text.

The stepwise restriction of parameters to a common one for all herbicide treatments revealed that solely the influence of weed density in autumn on TFI (coefficient *b*) was specific for the treatments (Fig. 5, left). The coefficient *b* decreased in the order DSSstd > DSSred ≈ AdvLoc > AdvSer, while the corresponding *P*‐values increased in the same sequence. Weed density significantly affected herbicide intensity exclusively in the DSS treatments. Weed density had a direct, strongly significant positive effect on species richness in early summer, with a similar effect across all decision‐making paths (coefficient *e* = 1.21; Fig. 5, left). A second pathway from weed density to diversity was mediated by herbicide intensity, with indirect effects of −0.78, −0.41, −0.35, and 0 for treatments DSSstd, DSSred, AdvLoc, and AdvSer, respectively (path b × path c). Consequently, the total effect of density on species richness was 0.43, 0.80, 0.86, and 1.21 for the same treatments (path b × path c + path e). The same trends were observed for the Shannon index (Fig. 5; right). High species richness in the autumn did not affect herbicide intensity (path a; Fig. 5, left). In contrast, a high Shannon index in autumn significantly decreased herbicide intensity (path a; Fig. 5, right). A direct significant effect of diversity in autumn on diversity in early summer was only found for species richness but not for the Shannon index (path d; Fig. 5).

#### 3.2.2 Different path effects (standardized coefficients)

In the path models of species richness, the standardized coefficients (Table 3, left) indicated a particularly strong direct influence of weed density on diversity in early summer (path e). These models also showed that the importance of individual paths changed in cropping systems with different ways of decision‐making: for example, in the DSS models, the influence of weed density on herbicide intensity (path b) exceeded the influence of weed density on diversity. For the Shannon index path models (Table 3, right), the standardized coefficients demonstrate the importance of high autumn diversity for low herbicide intensity (path a): considering the absolute values, the path a coefficient comes first (AdvSer), second (AdvLoc, DSSred), or third (DSSstd).

**Table 3:** Weed diversity–herbicide intensity path model: standardized path model coefficients and coefficients of determination (*R* ^2^).

↗	Path coefficients		Species richness				Shannon index			
	From	To	DSSstd	DSSred	AdvLoc	AdvSer	DSSstd	DSSred	AdvLoc	AdvSer
a	Diversity_autumn_	Herb. Intensity	−0.12	−0.22	−0.15	−0.24	−0.22	−0.37	−0.27	−0.37
b	Density_autumn_	Herb. intensity	0.73	0.67	0.40	0.00	0.70	0.53	0.33	−0.12
c	Herb. Intensity	Diversity_summer_	−0.40	−0.23	−0.30	−0.21	−0.19	−0.12	−0.15	−0.11
d	Diversity_autumn_	Diversity_summer_	0.32	0.33	0.28	0.33	−0.17	−0.17	−0.17	−0.16
e	Density_autumn_	Diversity_summer_	0.45	0.47	0.40	0.46	0.24	0.25	0.24	0.23
f	Diversity_autumn_	Density_autumn_	0.54	0.54	0.54	0.54	−0.02	−0.02	−0.02	−0.02
	*R* ^2^									
	Herbicide intensity		0.45	0.34	0.12	0.06	0.54	0.42	0.19	0.08
	Diversity_summer_		0.31	0.41	0.36	0.59	0.05	0.06	0.07	0.15

Diversity in autumn is based on counts, diversity in summer after herbicide treatments is based on biomass; herbicide intensity was determined as treatment frequency index (TFI). **↗**: letters in this column refer to arrows in Fig. 2.

DSSstd, decision support system standard; DSSred, decision support system with reduced target efficacy; AdvLoc, local advisor; AdvSer, advisory service.

For DSSstd, herbicide intensity TFI could be predicted from density and species richness to an *R* ^2^ of 45% and from density and Shannon index to 54% (*R* ^2^) (Table 3, bottom). Thus, this herbicide decision‐making offered a better explanation of the level of TFI than the other ways of decision‐making. Conversely, species richness for the DSSstd was worse explained (*R* ^2^ = 31%) than in the models with other ways of decision‐making. In particular, the *R* ^2^ of 59% in the AdvSer model was high (Table 3, bottom left). The *R* ^2^ for Shannon Indices in early summer was low in all models (*R* ^2^ ≤ 15%; Table 3, bottom right).

### 3.3 Weed species composition

**Figure 6:**
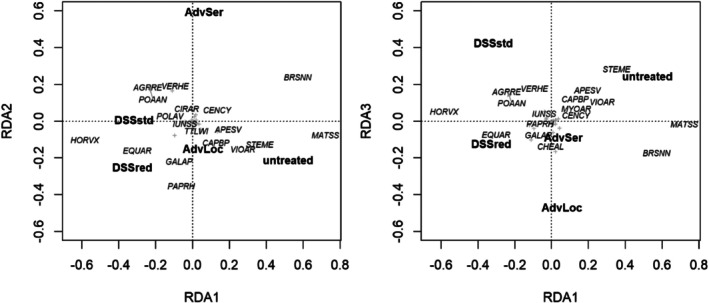
Ordination biplots (redundancy analysis (RDA), left: axes 1 and 2, right: axes 1 and 3) showing the effects of different ways of herbicide decision‐making on the weed species composition. DSSstd, decision support system standard; DSSred, decision support system advising reduced efficacies; AdvLoc, local advisor; AdvSer, official advisory service. Species names: AGGRE, *Elymus repens*; APESV, *Apera spica‐venti*; BRSNN, *Brassica napus*; CAPBP, *Capsella bursa‐pastoris*; CHEAL, *Chenopodium album*; CENCY, *Centaurea cyanus*; CIRAR, *Cirsium arvense*; EQUAR, *Equisetum arvense*; GALAP, *Galium aparine*; HORVX, *Hordeum vulgare*; IUNSS, *Iuncus* spec.; MATSS, *Matricaria* spec.; MYOAR, *Myosotis arvensis*; PAPRH, *Papaver rhoeas*; POAAN, *Poa annua*; POLAV, *Polygonum aviculare*; STEME, *Stellaria media*; TTLWI, *Triticale* (winter); VERHE, *Veronica hederifolia*; VIOAR, *Viola arvensis* (EPPO codes^34^).

The PERMANOVA revealed that the variation attributed to the experimental treatments was significant in both the analysis including the untreated control (*P*(pseudo‐F) = 0.002) and the analysis excluding the untreated control (*P*(pseudo‐F) = 0.016). The redundancy analysis arranged both DSS treatments on the first axis in opposition to the untreated control (Fig. 6). While the untreated control was characterized by *Matricaria* spec., *Viola arvensis*, *Capsella bursa‐pastoris*, *Stellaria media* and volunteer oilseed rape, species associated with the DSS treatments were mainly grasses (*Elymus repens* and *Poa annua*) and volunteer winter cereals. For DSSred, there was also a correlation with the occurrence of *Galium aparine* and *Papaver rhoeas*. These two species were negatively correlated with AdvSer on the second axis (Fig. 6, left) The third axis indicates that the species composition in the AdvLoc treatment differed from that in the untreated control (Fig. 6, right).

For the prominent species identified in the ordination, Table 4 lists the biomass in absolute values and relative to the untreated control. Species that correlated with any herbicide treatment never had more biomass than in the untreated control. The grasses strike with low biomass reduction compared to the untreated control, *Poa annua* (64–71% in the DS systems) and *Elymus repens* (40–88% in the DSSs and AdvSer), while these two species were reduced to 21% and 5%, respectively, in the AdvLoc treatment (Table 4). However, the absolute biomass of these two grasses was lower than that of the dicot species and the grass *A. spica‐venti*. The high biomass of volunteer barley was not reduced by more than 51% in any of the treatments (Table 4).

**Table 4:** Biomass (dry mass) of selected species in 15 winter wheat field trials in the untreated control and after herbicide treatment due to different herbicide decision making.

Species	Untreated	DSSstd		DSSred		AdvLoc		AdvSer	
	g m^−2^	g m^−2^	%	g m^−2^	%	g m^−2^	%	g m^−2^	%
*Apera spica‐venti*	4.00	0.11	3	0.12	3	0.02	0	0.02	0
*Brassica napus*	16.18	0.11	1	0.19	1	1.25	8	0.48	3
*Capsella bursa‐pastoris*	7.09	0.13	2	0.14	2	0.03	0	0.00	0
*Elymus repens*	0.96	0.84	88	0.38	40	0.05	5	0.57	60
*Galium aparine*	12.58	0.26	2	1.08	9	0.59	5	0.32	3
*Hordeum vulgare*	13.01	9.96	77	7.22	56	9.41	72	6.32	49
*Matricaria* spec.	40.21	1.07	3	0.39	1	2.12	5	0.28	1
*Papaver rhoeas*	17.10	0.43	3	1.65	10	0.82	5	0.07	0
*Poa annua*	0.42	0.30	71	0.27	64	0.09	21	0.15	35
*Stellaria media*	15.56	0.95	6	0.02	0	0.09	1	0.14	1
*Viola arvensis*	21.38	1.42	7	0.42	2	2.97	14	0.50	2

Mean values and percentages of the untreated control.

DSSstd, decision support system standard; DSSred, decision support system advising reduced efficacies; AdvLoc, local advisor; AdvSer, advisory service.

## 4 DISCUSSION

### 4.1 Does using a DSS help to maintain weed species diversity?

The expectation that herbicide applications based on the CPO DSS would lead to more diverse weed communities was unmet. Alternatively, it can be stated that a local consultant who surveyed the fields before the weed control will not force the weed diversity loss in the arable fields any more than the DSS. This result fits the fact that herbicide intensity did not differ between treatments with DSSstd and AdvLoc, and the management success as measured by weed biomass was also the same. In contrast to the results here, other research has shown a significant reduction in TFI after CPO treatment.^11^, ^13^, ^35^ In these settings, using the CPO may also provide diversity benefits. Only the standard AdvSer treatment resulted in a stronger lowering effect on biomass and diversity parameters, although herbicide intensity was not significantly increased even in this experimental treatment. However, Wuepper *et al*. found that the public extension service, corresponding to the AdvSer in our study, resulted in more preventive measures and reduced insecticide use by fruit growers.^36^ The difference in the present study on herbicide use is probably not due to the difference between public/private advice but to advice without or with an on‐site inspection and assessment of the weeds in the field. The result suggests that more selective herbicides are chosen when the weed growth in the field is assessed visually before treatment.

The Hill's series showed that no weed management is not necessarily beneficial to diversity. The species richness benefited from the absence of herbicide treatments. Individual species, however, became more dominant, as the Berger–Parker index showed. This is only partly consistent with the results of Grundy *et al*., who found in a 9‐year weed management trial in vegetable cultivation that species richness increased with a lower herbicide rate, but species dominance decreased.^16^ Different species composition, other herbicides and environmental effects (e.g., weather) may have led to the deviating results. However, we underline that simply reducing the use of herbicides does not automatically increase weed diversity. Targeted management may be more successful in preventing the dominance of individual species.

Species composition is equally important to farmers and ecologists. Those species that were ordered by ordination toward the untreated control are dicotyledonous weeds found in intensive winter oilseed rape – winter cereal crop rotations. These species were well represented in the weed flora of the autumn surveys. The fact that the centroids of the DSSs were arranged in opposition to untreated on the first axis suggests that these species were controlled quite specifically. In this respect the species‐specific control of DSS was successful. However, the fact that monocotyledonous weeds now appear associated with the DSS plots must be viewed critically. The RDA considers weed densities in relative terms; however, even in absolute values, *A. spica‐venti*, *Poa annua* and *Elymus repens* are more present in the DSS treatments (DSSstd, DSSred) than the advisor managed (AdvLoc, AdvSer). Monocotyledonous plants are particularly feared in cereal‐rich crop rotations, as the choice of herbicides is limited, and the risk of developing herbicide resistance is high.^37^ This article is based on 15 one‐year trials, in these weeds were counted after treatments and before harvest. Only surviving weeds are able to produce seeds and thus cause a shift in the weed species composition. As the herbicide recommendations in the following years will react on differences in the weed flora, it would be interesting to see how the species composition moves in the long term, for example, by analysing trials with different decision making over many years.

### 4.2 How do biodiversity and herbicide intensity influence each other?

We introduced the state of the art, that a low diversity of species leads to a high herbicide intensity. Theoretical considerations suggest that a greater diversity of species lead to a lower herbicide intensity: In the course of selection of weed species, their diversity decreases. Only those species that are more tolerant to the control, for example by herbicides, withstand the selection pressure. Therefore, their proportion increases in the selection process over many years. This less herbicide‐sensitive species community requires a higher herbicide intensity for control. However, with some justification, the opposite hypothesis can also be put forward: with a large number of different species, it is unlikely that one herbicide or even a single herbicidal active ingredient will control all species. Thus, if the number of species increased, a higher number of herbicides would also be needed. To be effective, these must each be applied at certain minimum rates so that herbicide intensity would increase with increased diversity.

In the structure of the system of diversity and herbicide intensity, as visualized in Figs 2 and 5, two submodels, that is, groups of paths, can be identified. While paths d, e, and f represent ecological relations, paths a, b, and c represent agricultural relations as herbicides are involved. In the latter, farmers can intervene if they want to change the system. It was expected that the ecological interrelationships would be independent of the type of decision‐making. The same common parameters for paths d, e and f, resulting from selecting the path model, confirmed this. The highly significant correlation between density and species richness in the autumn stands for the positive correlation of an individual‐based species accumulation curve: higher densities increase the probability of another species in the sample. As we have shown, it can be assumed that a high density and species richness in the autumn indicates a high species richness in the summer. Winter‐annual weeds can survive because herbicide control did not reach a 100%, for example, due to spraying shadows by plant leaves. Therefore, higher diversity and higher density in autumn may lead to higher diversity and higher density in summer, although we did not measure it. With higher density, the argument of the probability of detecting another species in the sample applies again. The soil seed bank can also be involved; Rotchés‐Ribalta *et al*. demonstrated an influence of the intensity of agrochemical use on the soil seed bank similar to the aboveground growing arable weeds.^38^ Thus, a seed bank that is generally species‐diverse and may enable winter‐annual and summer‐annual species to emerge.

The only path with significantly different parameters for the different ways of decision‐making in the agronomic submodel was the influence of weed density in the autumn on herbicide intensity. This influence is unsurprising since the DSSs are designed to include weed density in their recommendation. The influence of the density is limited by the fact that different species are weighted differently in herbicide selection and dosing. Functional species composition (function in the sense that the same monetary disadvantages to the crop are expected) appears to be similar enough among the 15 fields studied that the influence of density on TFI becomes evident in the DSS models. One consequence of the differing influence of density on TFI is that the overall effect of density on diversity in summer decreases in the order AdvSer > AdvLoc > DSSred > DSSstd. This implies a partial disentanglement of density in the autumn from diversity in the summer for the DSSs. This effect is highly desirable, as it allows agroecological maintaining high biodiversity while agronomically keeping weed densities low.

Different metrics of diversity focus on different problems. Species richness is related to species conservation. One or two individuals of a species can be sufficient for reproduction and save the species from extinction. The true diversity, which weights each species according to its density, also relates to the field ecosystem's functioning. This is because a species has to occur at a minimum density to perform an agricultural and ecological function in a field. The path model for the Shannon index does not show any significant relationships in the ecological submodel (paths d, e and f). This may be due to species‐specific characteristics in combination with our approach of calculating summer diversity based on biomass. Thus, species in which the individual builds a lot of biomass contribute quite differently to diversity in summer than in autumn. In the agronomic submodel (paths a, b and c), the hypothesis that increased diversity leads to decreased herbicide use is supported. Generalizing this result should still be done cautiously. Weed species in general occur spatially variable. Although we sampled weeds of 4 m^2^ per treatment, the infestation of the total field can always deviate from that assessed in plots. In addition to diversity, different species composition plays a role, again because of species‐specific characteristics, namely the ability of the weed to compete with the crop and the species and herbicide‐specific dose–response relationships.

### 4.3 Conclusion

In general, the question is: what kind of diversity do we as a society want? Once we have developed these objectives and agreed on a ‘target biodiversity’ with farmers and ecologists, we have to ask whether chemical weed control is a possibility for specific regulation at all. That means, are our herbicides specific enough to spare desired species while sufficiently controlling others? We have demonstrated that applying herbicide DSSs aimed at economic benefit do not automatically lead to a greater weed diversity. This does not mean, however, that other systems that aim directly to promote diversity would not be suitable. Computer‐based DSSs might also be useful for this objective in order to simplify the complexity of the decision for the user. However, such systems would still need to be developed.

## Data Availability

Research data are not shared.
